# A probabilistic view of protein stability, conformational specificity, and design

**DOI:** 10.1038/s41598-023-42032-1

**Published:** 2023-09-19

**Authors:** Jacob A. Stern, Tyler J. Free, Kimberlee L. Stern, Spencer Gardiner, Nicholas A. Dalley, Bradley C. Bundy, Joshua L. Price, David Wingate, Dennis Della Corte

**Affiliations:** 1 Department of Computer Science, Brigham Young University, Provo, UT USA; 2 Department of Chemical Engineering, Brigham Young University, Provo, UT USA; 3 Department of Chemistry and Biochemistry, Brigham Young University, Provo, UT USA; 4 Department of Physics and Astronomy, Brigham Young University, Provo, UT USA

**Keywords:** Computational models, Machine learning, Protein design

## Abstract

Various approaches have used neural networks as probabilistic models for the design of protein sequences. These "inverse folding" models employ different objective functions, which come with trade-offs that have not been assessed in detail before. This study introduces probabilistic definitions of protein stability and conformational specificity and demonstrates the relationship between these chemical properties and the $p(\text {structure}|\text {seq})$ Boltzmann probability objective. This links the Boltzmann probability objective function to experimentally verifiable outcomes. We propose a novel sequence decoding algorithm, referred to as “BayesDesign”, that leverages Bayes’ Rule to maximize the $p(\text {structure}|\text {seq})$ objective instead of the $p(\text {seq}|\text {structure})$ objective common in inverse folding models. The efficacy of BayesDesign is evaluated in the context of two protein model systems, the NanoLuc enzyme and the WW structural motif. Both BayesDesign and the baseline ProteinMPNN algorithm increase the thermostability of NanoLuc and increase the conformational specificity of WW. The possible sources of error in the model are analyzed.

## Introduction

The inverse folding protein design problem is to design a protein that folds into a structure with desirable properties or function (thermostability, binding affinity, enzymatic activity, etc.)^1^. The ability to design proteins with desired properties is key to designing new protein-based medicines, materials, and nanotechnology. Rational design allows humans to incorporate chemical knowledge into designs^2^, but manual approaches limit the sequence search space to human efficiency and knowledge of chemical principles. Computational protein design allows the user to quickly screen millions of sequences and to automate decisions based on an “objective function” that mathematically quantifies the quality of a protein design. In computational protein design, the choice of an objective function is essential in linking the computational design algorithm to a desired chemical function or property^3^.

One common protein sequence design objective is to find the minimum-energy sequence for a desired protein structure^4^. This approach has been used since the first Rosetta energy function was introduced^5, 6^, continuing into current usage with improved energy functions^7^. Over the years, a number of algorithms have been developed to find the minimum-energy sequence for a given structure^8–12^.

However, finding the minimum-energy sequence for a given structure does not guarantee that the designed sequence folds into the desired structure. As observed by^4^, minimizing the absolute energy of the sequence for the structure can result in sequences with energy minima at different conformations. To address this^4^, uses gradient descent over sequence space to maximize $p(\text {structure}|\text {seq})$, such that predicted structure matches target structure, using trRosetta^13^ as a model for $p(\text {structure}|\text {seq})$.

Several subsequent works attempt to maximize the improved, $p(\text {structure}|\text {seq})$ objective. ColabDesign^14, 15^ uses an input optimization method similar to^4^ but with AlphaFold2^16^ as the structure prediction model. Similarly,^17^ uses trRosetta as a model for $p(\text {structure}|\text {seq})$ but uses Markov Chain Monte Carlo (MCMC) optimization over sequences to maximize the Kullback-Leibler divergence between $p(\text {structure}|\text {seq})$ for a designed sequence and $p(\text {structure}|\text {seq})$ for a “background” structure.

However, the authors of both^4^ and^17^ found that optimizing over the inputs of a forward structure prediction model leads to “adversarial” sequences that, while predicted to fold to the target structure, are insoluble in practice^15, 18^. This is a known problem exploited in “adversarial optimization”, where optimizing over the inputs to a model identifies inputs for which the model has high confidence, but which do not lie on the manifold of data for which the model makes reliable predictions^19^.

There have been several attempts to resolve the problem of insoluble adversarial sequences. ^4^ and^17^ introduce terms to the original objective to increase the probability that sequences fall on the manifold of training data for the structure prediction model. ^18^ attempts to resolve the adversarial problem by using ProteinMPNN (which is trained with a $p(\text {seq}|\text {structure})$ objective) to redesign protein sequences for backbones designed by^17^. But all of these solutions alter the original design objective such that designed sequences are different from the sequences that maximize $p(\text {structure}|\text {seq})$.

In this work, we argue for a return to the $p(\text {structure}|\text {seq})$ objective function by theoretically linking this objective to notions of protein stability and conformational specificity^20^. Stability describes the energy difference between the folded native state and the unfolded denatured state of a protein. Conformational specificity refers to how strongly a protein prefers the native state over other folded conformations.

We present formal probabilistic definitions of protein stability and conformational specificity which illustrate the relationship between these protein design criteria and the $p(\text {structure}|\text {seq})$ objective. The link to protein stability and conformational specificity offers experiments that can be used to verify designs and motivates careful adherence to the $p(\text {structure}|\text {seq})$ objective function when stability and/or conformational specificity is the design goal.

**Figure 1:**
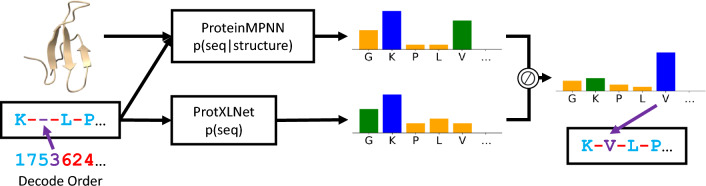
The BayesDesign model for de novo protein design.

Next, we describe “BayesDesign”, a new sequence design algorithm to maximize the $p(\text {structure}|\text {seq})$ objective without relying on gradient descent or MCMC optimization techniques, thereby avoiding adversarial inputs to a $p(\text {structure}|\text {seq})$ model. Our approach uses existing models for $p(\text {seq}|\text {structure})$ and $p(\text {seq})$ and applies Bayes’ Rule to design proteins that maximize $p(\text {structure}|\text {seq})$ (see Fig. 1).

Finally, we evaluate the stability and conformational specificity of BayesDesign-designed proteins on two model systems: the luminescent NanoLuciferase (NanoLuc) enzyme and the WW beta sheet motif.

Our contributions are as follows:

- We mathematically formalize protein design objectives for protein stability and conformational specificity and show how they relate to the Boltzmann probability objective function $p(\text {structure}|\text {seq})$.
- We derive a tractable probabilistic model, “BayesDesign” to design protein sequences maximizing the Boltzmann probability objective $p(\text {structure}|\text {seq})$ without finding adversarial sequences.
- We show that sequences designed by the BayesDesign algorithm increase the stability and conformational specificity of proteins structures relative to the native sequences corresponding to those structures.

## Theory

### Boltzmann probability, stability, and conformational specificity

The probability $p(\text {structure}|\text {seq})$ is known as the Boltzmann probability of a protein conformation and relates the Gibbs free energy of a state to the probability of a protein existing in that state.

$$\begin{aligned} \quad p_{boltz}&= p(\text {structure}=X|\text {seq}=s) \\&=\frac{e^{-G(X) / kT}}{\sum _{C\in \mathcal {C}}e^{-G(C) / kT}} \end{aligned}$$

SI Section A.1 introduces probabilistic definitions of protein stability and conformational specificity and shows that both are subsets of the Boltzmann objective. This shows that maximizing the Boltzmann objective can be expected to increase stability and/or conformational specificity of designed proteins. The effect of this objective function on these desirable properties motivates the use of the $p(\text {structure}|\text {seq})$ objective and points to wet-lab experiments that can measure the effects of maximizing the Boltzmann probability objective.

### Bayes’ rule to maximize $p(\text {structure}|\text {seq})$

BayesDesign is a probability model and an algorithm to maximize $p(\text {structure}|\text {seq})$ without using gradient-based or MCMC optimization over the inputs of a deep neural network. We use Bayes’ Rule to rewrite the $p(\text {structure}|\text {seq})$ objective:

$$\begin{aligned}&\underset{s \in S}{{\text {argmax}}} \quad p(\text {structure}=X|\text {seq}=s) \\&\quad = \underset{s \in S}{{\text {argmax}}} \quad \frac{p(\text {seq}=s|\text {structure}=X)p(\text {structure}=X)}{p(\text {seq=s})} \\&\quad = \underset{s \in S}{{\text {argmax}}} \quad \frac{p(\text {seq}=s|\text {structure}=X)}{p(\text {seq=s})} \\ \end{aligned}$$

where the second step follows from the fact that $p(\text {structure}=X)$ does not depend on the sequence *s*. This allows us to maximize $p(\text {structure}=X|\text {seq})$ by using two models, $p(\text {seq}|\text {structure}=X)$ and $p(\text {seq})$. We use ProteinMPNN^18^ as a model for $p(\text {seq}|\text {structure}=X)$ and ProtXLNet^13^ as a model for $p(\text {seq})$. This allows us to adopt sequence decoding schemes similar to those commonly used in language models instead of optimizing over sequences. This approach yields faster sequence design while avoiding adversarial optimization issues. The choices of ProteinMPNN and ProtXLNet and the decoding algorithm are described in SI section A.2.1. We note that one limitation of applying Bayes’ Rule is that it relies on the argmax operator and thus requires some form of a greedy decoding scheme, making it unsuitable for sampling schemes commonly used in autoregressive inverse folding models.

## Experimental methods

As shown in the theory section, the Boltzmann probability objective is linked to protein stability and conformational specificity. An additional goal was to design soluble sequences, avoiding the insoluble adversarial sequences that plague other approaches using the Boltzmann probability objective. Thus, we evaluate designed sequences for stability, conformational specificity, and solubility.

We additionally validate designed sequences by comparing their AlphaFold-predicted structures to the AlphaFold-predicted structure of the wild type sequence^16^.

### Evaluation of stability and solubility for NanoLuc

We compare the stability and solubility of the wild type NanoLuc enzyme to sequences designed by the BayesDesign and ProteinMPNN algorithms.

NanoLuc has been heavily engineered to increase its enzymatic activity. ^21^ mutated 16 residues close to the active site, resulting in an increase in enzymatic activity by several orders of magnitude. This engineered sequence, with an added N-terminal Strep tag, is referred to in this work as the wild type Nanoluc. Using BayesDesign and ProteinMPNN, we redesign the enzyme while conserving various combinations of the active site and engineered residues, shown in Figure S5 and Table S1/S2. We evaluate the change in stability and solubility relative to the wild type sequence.

In vitro validation experiments are conducted using cell-free protein synthesis (CFPS), a versatile expression system that is well-suited for experimental in vitro investigations of de novo designed proteins^22–24^. CFPS is used in this work to obtain temperature-dependent protein solubility, which can be used as an indication of relative thermodynamic stability^25–27^, though it is important to note that protein solubility does not guarantee that a particular structural conformation is achieved. The BayesDesign mutants, the ProteinMPNN mutants, and the wild type NanoLuc are synthesized in vitro.

The expressed proteins are aliquoted and samples are heat treated at temperatures ranging from 37 to $95\,^{\circ }$C. After heat treatment, the samples are centrifuged, and soluble Nanoluc protein in the supernatant is measured. Experimental methods are detailed in the supplementary information.

### Evaluation of conformational specificity for WW

We also evaluate the conformational specificity of designed sequences by redesigning the WW domain of human protein Pin 1.

The structure of this short 34-residue peptide has been widely characterized^28^ and it is known to follow a two-state model of unfolding. In this study we use the folding reversibility of WW as a proxy for conformational specificity. Sequences with high conformational specificity tend to refold to the native conformation after heat treatment (i.e. are reversible); others fail to recover the native state due to falling into alternate conformations upon refolding^29^. WW is chosen due to its poor reversibility, as low as 40% in some design studies^30^. We redesign the WW sequence with BayesDesign with the hypothesis that maximizing the Boltzmann probability objective will increase the conformational specificity of the native state.

We evaluate conformational specificity via the reversibility of the circular dichroism (CD) spectrum. CD measures the wavelength spectrum over a heating and cooling process. WW shows a characteristic peak at 227 nm that disappears upon thermal denaturation. We evaluate the reversibility of WW (and designed sequences) by measuring the percent of the starting CD signal at 227 nm recovered after equilibration at $95\,^{\circ }$C and cooling back to $25\,^{\circ }$C as in^31^.

We compare the reversibility of the wild type sequence, a sequence designed by BayesDesign, and a sequence designed by ProteinMPNN. The rationale for using ProteinMPNN as a baseline is to compare the reversibility of sequences designed using the probability ratio of ProteinMPNN and XLNet (i.e. BayesDesign) versus sequences designed using the probabilities of ProteinMPNN alone.

Solid-phase peptide synthesis is used to synthesize the native WW sequence, the BayesDesign design, and a design by ProteinMPNN (see Tables S3 and S4 and Figure S16 for sequences and characterization).

## Results

As preliminary validation, we evaluated AlphaFold-predicted structures for NanoLuc wild type and designed sequences. AlphaFold-predicted structures for designed sequences aligned closely with the predicted structure for the wild type sequence (see Figure S4).

**Figure 2:**
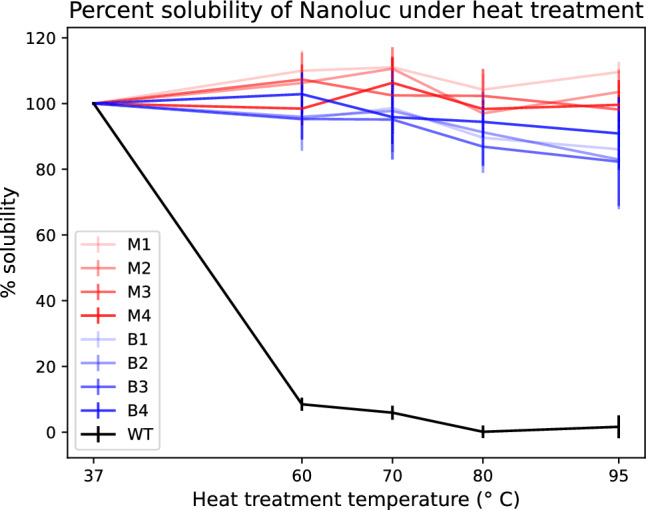
Experimental NanoLuc thermal stability comparing the wild type NanoLuc to mutants designed with ProteinMPNN (M1–M4) or BayesDesign (B1–B4). Each protein was expressed with a cell-free protein synthesis system at $37\,^\circ$C. Parallel aliquots were subjected to 15-min heat treatments at 37, 60, 70, 80, or $95\,^\circ$C and the remaining soluble protein was measured. The solubility data is the average of n = 3 biological replicates and the standard deviation error bars are shown as vertical lines. Interpolating lines show the temperature-dependent stability trend for each protein mutant.

While the hand-engineered wild type NanoLuc showed increased stability relative to the original luciferase enzyme^21^, both the BayesDesign and ProteinMPNN methods used in this study afforded a further increase in stability (Fig. 2). Remarkably, the BayesDesign and ProteinMPNN sequences retain solubility after heat treatment at $95\,^{\circ}$ C. This shows that in silico methods considering the full sequence when redesigning a protein can offer a significant increase in stability. Deep learning approaches like these make it possible to quickly redesign the entire protein sequence to increase stability, while taking advantage of stability patterns learned from data. The observed stability increases are particularly notable in the context of a reported 2% success rate of random substitution mutants to increase stability^32–36^.

Sequence designs that improve stability often do so at the expense of solubility^36^. Protein solubility is a desirable, if not essential, characteristic of active proteins for industrial, medicinal, and other applications^26, 36, 37^. From a biochemical perspective, randomly generated protein sequences are rarely soluble^38^ and random substitution mutations typically decrease protein solubility^36^. From an optimization perspective, previous gradient-based approaches to optimizing the $p(\text {structure}|\text {seq})$ objective often resulted in insoluble sequences^18^, requiring modification of the objective in order to obtain soluble sequence designs^4^.

**Figure 3:**
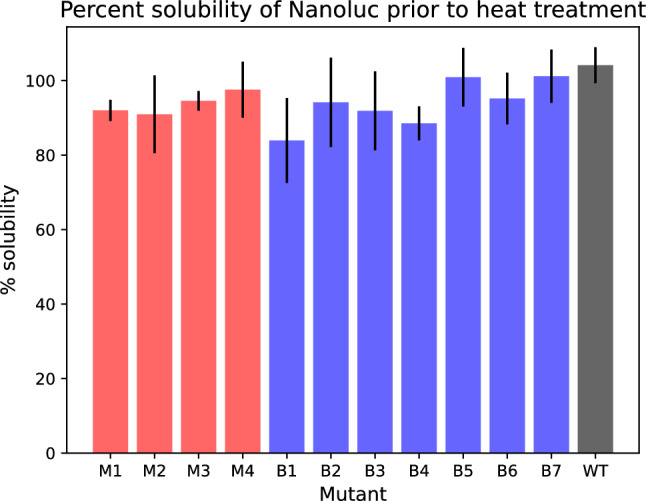
All NanoLuc mutants were found to be at least 84% soluble.

All designed sequences in this work (M1–M4 and B1–B4) have baseline solubility greater than 84% when expressed at $37^{\circ }$ (see Fig. 3). To our knowledge, this work is the first to present soluble protein designs using an algorithm that optimizes the unmodified $p(\text {structure}|\text {seq})$ objective.

These stabilization results come with an important caveat: the BayesDesign (B1–B4) and ProteinMPNN (M1–M4) sequences removed all appreciable enzymatic activity of NanoLuc, as shown in Figure S8. This points to the potential pitfalls of redesigning proteins whose function is sensitive to changes in structure and flexibility. First, changing a large number of residues is more likely to change a residue essential to protein function. Second, the theory introduced in section A.1.1 shows that the Boltzmann probability objective is linked to stability and conformational specificity, which may limit the flexibility needed to attain a catalytic conformation. The elimination of enzymatic activity indicates that the Boltzmann probability objective is not well suited for designing enzymes whose function depends on flexibility.

As an additional investigation, it was hypothesized that a BayesDesign sequence could preserve some enzymatic activity if fewer residues were mutated. To this end, the BayesDesign method was used to design sequences B5, B6, and B7 which have only two amino acid substitution mutations. These sequences exhibit greater thermal stability than the wild type enzyme (Figure S9A), but the enzymatic activity decreased by 500-fold or more (Figure S9B). Furthermore, the extent of stabilization correlated with the extent of activity lost for the BayesDesign sequences B5, B6 and B7 (Figure S9C). Taken together, these results support the theory of a theoretical tradeoff between stability and activity of the Nanoluc Enzyme. Future enzyme design studies are needed to further explore this tradeoff.

We also evaluated the BayesDesign algorithm on the short peptide WW. AlphaFold validation of BayesDesign and ProteinMPNN-designed sequences showed close agreement between ProteinMPNN and the wild type structures and partial agreement between the BayesDesign structure and the wild type (see Figure S11).

**Figure 4:**
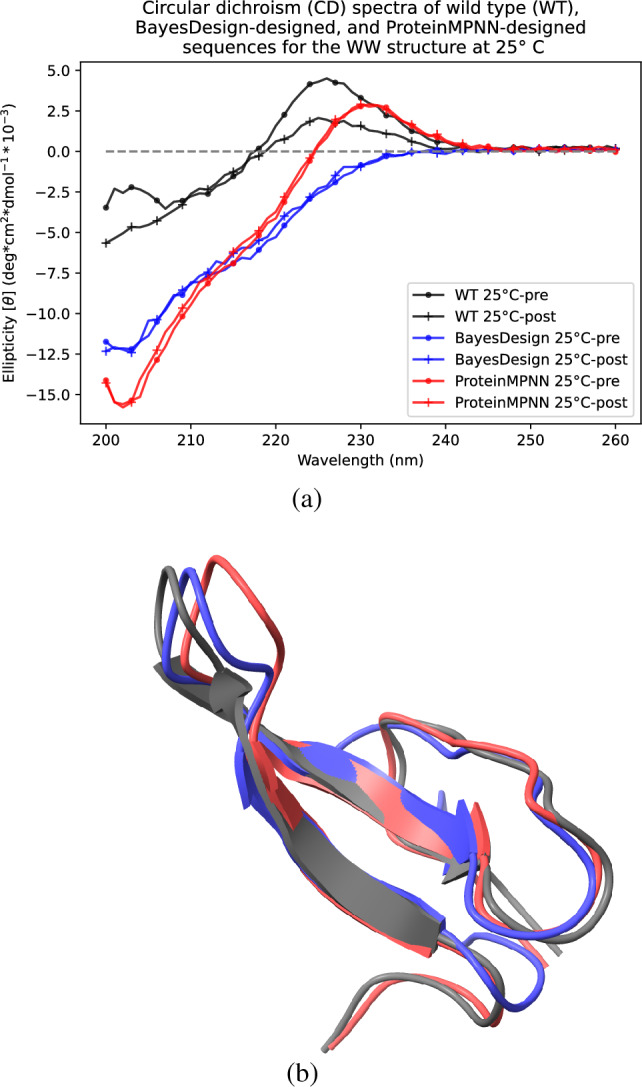
CD spectra indicating reversibility of BayesDesign and ProteinMPNN folding and corresponding structures. (**a**) Circular dichroism (CD) profile of wild type (black), BayesDesign (blue), and ProteinMPNN (red) sequences at $25\,^{\circ }$C before and after heat treatment. ProteinMPNN and BayesDesign both achieve high reversibility, but neither has the $\sim$227 nm peak that distinguishes the native WW structure. (**b**) Protein structures from molecular dynamics frames that most closely match the observed CD spectra for the WW wild type (black), BayesDesign (blue), and ProteinMPNN (red) sequences.

We next used CD to evaluate the reversibility of WW folding. However, a wavelength scan at $25\,^\circ$C revealed that neither ProteinMPNN nor BayesDesign sequences had the characteristic peak at 227 nm like that of WW (see Fig. 4). Due to this discrepancy, we ran molecular dynamics simulations to further evaluate how similar the BayesDesign and ProteinMPNN structures were to the wild type structure. We used the PDBMD2CD tool^39^ to predict CD spectra from evenly-spaced frames of the molecular dynamics simulation, then selected the frames whose predicted spectra most closely matched the observed spectra for each of the BayesDesign, ProteinMPNN, and wild type structures (see Figure S15). We then measured the RMSD between the structures of the designed sequences and the structure of the wild type sequence (see Fig. 4). We found that the RMSD between BayesDesign and the wild type was 3.01 Å, and the RMSD between ProteinMPNN and the wild type was 1.18 Å, indicating that the structures did closely match the wild type.

Heating the BayesDesign peptide to $95\,^\circ$C showed an ellipticity shift of the minimum at 203 nm, indicating some denaturation. After cooling, the minimum at 203 nm was completely recovered, showing that the BayesDesign peptide was 100% reversible for its adopted conformation at its minimum of 203 nm (see Figure S13) whereas native WW was only 56% reversibile at its 227 nm peak (see Figure S12). The high reversibility of the BayesDesign peptide relative to the wild type confirms the hypothesis that using the BayesDesign algorithm results in designs with high conformational specificity. Interestingly, ProteinMPNN also demonstrated high reversibility (see Figure S14).

## Discussion

In this study we provide theory suggesting that a protein designed to optimize the Boltzmann probability objective should increase either stability, conformational specificity, or both, relative to a sequence not optimized to maximize Boltzmann probability. We tested this hypothesis by redesigning WT NanoLuc and evaluating its stability via temperature-dependent protein solubility, and redesigning WT WW and evaluating its conformational specificity via CD reversibility.

We found that BayesDesign NanoLuc increased stability relative to WT NanoLuc, though at the cost of enzymatic activity. BayesDesign WW had high conformational specificity relative to WT NanoLuc, though with less fidelity to the WT structure than ProteinMPNN. These findings support the use of the $p(\text {structure}|\text {seq}$) objective as used by BayesDesign, and also point to several possible sources of error to consider when developing probabilistic algorithms for protein design.

### Source of error 1: using the wrong objective function

The objective function used to train the probability model should mimic the real-world effects of interest. In this study, we described three probabilistic objectives: the protein stability objective, the conformational specificity objective, and the Boltzmann probability objective. Proteins that maximize these objective functions can be expected to maximize stability, specificity, and Boltzmann probability, respectively.

These are three possible design objectives, but they may not be the best objective functions to model the desired outcome of some design tasks. For example, if the goal is to increase enzymatic activity, Boltzmann probability may be the wrong objective because both stability and conformational specificity limit the flexibility of enzymes, which is often key to their function.

### Source of error 2: using the wrong probability model for the objective

Even if trained with the correct objective function, a probability model may be incorrect. If the model is trained on data, biases in the training data may limit the accuracy of the model on the desired task. BayesDesign consists of two models, ProteinMPNN and ProtXLNet. ProteinMPNN was trained on static protein crystal structures in the PDB. ProtXLNet was trained on the UniRef100 database^40^. Both of these models capture information about the joint probability of protein sequences and structures. Within that joint distribution, ProteinMPNN predicts sequence conditioned on structure, and ProtXLNet predicts sequence marginalized (summed) over all structures.

However, the PDB is biased toward crystallizable sequences, meaning that the marginal distribution *p*(*seq*) for the PDB is different from that of UniRef100. We hypothesize that this miscalibration is likely why BayesDesign fails to design proteins more stable or with higher conformational specificity than ProteinMPNN.

The adversarial sequences discovered by^4^ and^17^ are another example of design problems arising from an incorrect probability model. The forward probability model $p(\text {structure}|\text {seq})$ was incorrect in the regime of low-probability sequences, resulting in designed sequences predicted to fold into structures with high probability, but which were insoluble in practice.

### Source of error 3: using a poor optimization scheme for the probability model

Given an objective function that matches the desired real-world phenomena and an accurate probability model for that objective function, the task remains to find the protein sequence that optimizes the objective function under the probability model.

However, optimizing over a sequence space of size $20^L$ requires approximation for large proteins. For this reason, various algorithms use optimization schemes that seek to navigate the loss surface more efficiently. The approximations involved in these optimization schemes often result in designs that do not identify the global optimum.

The greedy decoding used for BayesDesign and ProteinMPNN in this paper optimizes over each amino acid position sequentially by selecting each amino acid position conditioned on all previously-selected positions. This does not exhaustively search the joint distribution of all amino acid sequences. However, we find that sequences designed with greedy decoding over the BayesDesign model achieve higher probability on the BayesDesign model than sequences designed by ProteinMPNN, and vice-versa.

## Conclusion

We introduce a probabilistic framework for predicting the effects of the Boltzmann probability objective function $p(\text {structure}|\text {seq})$ on protein stability and conformational specificity. We introduce BayesDesign, a new protein design algorithm to maximize this design objective by applying Bayes’ rule to unconditional and structure-conditioned autoregressive sequence models.

Compared to other methods that maximize $p(\text {structure}|\text {seq})$, our method designs soluble sequences without modifying the original objective function. Compared to methods that maximize $p(\text {seq}|\text {structure})$, we have theoretical reasoning that suggests that designed sequences should increase protein stability and/or conformational specificity.

We show that redesigning the NanoLuc enzyme with this objective increases its stability. For the WW peptide, the BayesDesign-designed sequence achieves higher conformational specificity than the wild type peptide.

One of the surprising results of this study was the strong performance of ProteinMPNN relative to BayesDesign, despite it lacking the theoretical guarantees afforded by BayesDesign. We identify several possible reasons for this discrepancy and suggest improvements to address in future work.

### Supplementary Information

Supplementary Information.

## Data Availability

Code for the BayesDesign algorithm is available at https://github.com/dellacortelab/bayes_design. Experimental data is available from the corresponding author upon request.
