# Supplementary material for: A probabilistic view of protein stability, conformational specificity, and design
Source: Sci Rep. 2023 Sep 19;13:15493. doi: 10.1038/s41598-023-42032-1 (PMC10509192; doi:10.1038/s41598-023-42032-1)
Supplement: Supplementary file 1 — Supplementary Information. [file 41598_2023_42032_MOESM1_ESM.pdf]

Supplementary Information

August 13, 2023

A Theory

A.1 Formalizing protein design objectives

A.1.1 Boltzmann probability

The **Boltzmann probability** of a protein conformation X relates its energy to its probability:

$$\begin{aligned} p_{boltz} &= p(\text{structure} = X | \text{seq} = s) \\ &= \frac{e^{-G(X)/kT}}{\sum_{C \in \mathcal{C}} e^{-G(C)/kT}} \end{aligned}$$

where $\mathcal{C}$ is the set of all possible states and $G(C)$ refers to the Gibbs free energy of a state C. This is the objective function described in [Norn et al., 2021]. The denominator can be partitioned into three terms:

$$p_{boltz} = \frac{e^{-G(X)/kT}}{e^{-G(X)/kT} + e^{-G(Y)/kT} + e^{-G(Z)/kT}}$$

where Y is the set of alternate folded conformations, and Z is the set of denatured conformations. The rationale for partitioning the denominator into these three terms is given in section A.1.4.

For notational simplicity throughout this paper, we write $G(X)$ to refer to the Gibbs free energy of a structure X conditioned on its corresponding sequence s.

A.1.2 Protein stability

Protein stability is defined as the Gibbs free energy difference between the denatured state and native state:

$$\Delta G = G(Z) - G(X)$$

where X is the minimum-energy folded state and Z is the denatured state.

Maximizing protein stability is equivalent to maximizing the probability ratio between the minimum-energy folded state X and the denatured state Z (see Proposition 1 in the SI):

$$\begin{aligned}
&\underset{s\in S}{\operatorname{argmax}} \quad \Delta G \\
&= \underset{s\in S}{\operatorname{argmax}} \quad G(Z|\text{seq}=s) - G(X|\text{seq}=s) \\
&= \underset{s\in S}{\operatorname{argmax}} \quad \frac{p(\text{structure}=X|\text{seq}=s)}{p(\text{structure}=Z|\text{seq}=s)}
\end{aligned}$$

where $G(X|\text{seq}=s)$ is the Gibbs free energy of the native state for a given sequence and $G(Z|\text{seq}=s)$ is the Gibbs free energy of the denatured state for a given sequence s.

Based on this equivalence, we propose a **probabilistic definition of protein stability** that is not equal, but which is maximized when the original definition of protein stability is maximized:

$$\begin{aligned}
p_{stb} &= \frac{p(\text{structure}=X|\text{seq}=s)}{p(\text{structure}=Z|\text{seq}=s)} \\
&= \frac{e^{-G(X)/kT}}{e^{-G(Z)/kT}}
\end{aligned}$$

Writing the definition of protein stability in its probabilistic form, we can see the relationship between protein stability and the Boltzmann probability objective - they differ only in the terms present in the denominator.

A.1.3 Conformational specificity

We also introduce a **probabilistic definition of conformational specificity** which serves as a compliment to the probabilistic definition of protein stability. A sequence that maximizes this metric maximizes the probability ratio between the native conformation and alternate folded conformations.

$$\begin{aligned}
p_{spc} &= \frac{p(\text{structure}=X|\text{seq}=s)}{p(\text{structure}=Y|\text{seq}=s)} \\
&= \frac{e^{-G(X)/kT}}{e^{-G(Y)/kT}}
\end{aligned}$$

Conformational specificity measures the tendency of a protein structure to maintain one conformation over other folded conformations. This is important, as proteins with high conformational specificity to a are less likely to adopt another conformation that may aggregate [Zhu et al., 2015], driving the dynamic equilibrium of conformations toward the undesirable conformation.

Whereas protein stability compares the probability of the native folded state to the denatured state, conformational specificity compares the probability of the native state to alternate folded conformations.

A.1.4 Relationship between Boltzmann probability, protein stability, and conformational specificity

If a sequence design approach assumes a model $p(\text{structure} = X|\text{seq})$ of the Boltzmann probability of a given conformation given a sequence, then maximizing that objective has predictable effects on protein stability and specificity.

These effects are made apparent by partitioning the denominator of the Boltzmann probability objective into three terms, as shown in section A.1.1. There are three ways to maximize $p(\text{structure} = X|\text{seq})$: 1) by reducing the Gibbs free energy $G(X)$ of the native state X, 2) by increasing the Gibbs free energy $G(Z)$ of the denatured state Z, or 3) by increasing the Gibbs free energy $G(Y)$ of alternate folded conformations.

Increasing $p(\text{structure} = X|\text{seq})$ by deepening the energy well of the structure X will increase both stability and conformational specificity. Increasing $p(\text{structure} = X|\text{seq})$ by increasing the energy of the denatured state Z will increase stability without affecting conformational specificity, and increasing $p(\text{structure} = X|\text{seq})$ by removing low-energy alternate conformations $y \in Y$ will increase conformational specificity without affecting stability.

Thus, assuming that the probability model is correct, maximizing $p(\text{structure} = X|\text{seq})$ will increase either the stability or conformational specificity of that state, and possibly both. However, it is possible that maximizing $p(\text{structure} = X|\text{seq})$ may increase stability at the cost of conformational specificity or vice versa, depending on which offers the greatest improvement to the Boltzmann probability.

Figure S1 shows a hypothetical case where the sequence that maximizes Boltzmann probability (Seq A) is different from the sequence that maximizes stability (Seq B) or conformational specificity (Seq C). Figure S2 illustrates a case (i.e. a set of energy landscapes) where optimizing Boltzmann probability would result in selecting the maximum stability sequence, and Figure S3 illustrates a case where optimizing Boltzmann probability would result in selecting the maximum specificity sequence.

A.2 Bayes' Rule to maximize $p(\text{structure}|\text{seq})$

A.2.1 Probabilistic models for $p(\text{seq}|\text{structure})$ and $p(\text{seq})$

The BayesDesign algorithm requires probability models of $p(\text{seq}|\text{structure})$ and $p(\text{seq})$. There are some properties that would be especially useful for these two models. First, they should be autoregressive models of the joint probability, enabling the use of greedy algorithms such as beam search for decoding. Additionally, it would be beneficial for the models to be order-agnostic probabilistic models, allowing for the selection of a decoding order that prioritizes the design of crucial sequence locations, such as those near a catalytic site.

We use ProteinMPNN [Dauparas et al., 2022] as a model for $p(\text{seq}|\text{structure})$ and ProtXLNet [Elnaggar et al., 2020] for $p(\text{seq})$. Both models possess the desired order-agnostic and autoregressive properties.

A.2.2 Decoding

The joint probability of a sequence can be factorized as the product of conditional probabilities using the chain rule of probability. This reduces to a ratio of probabilities for each position in the sequence, where at each position the probability ratio gives a score for each amino acid:

$$\begin{aligned}
&\underset{s\in S}{\operatorname{argmax}} \ \frac{p(\text{seq}|\text{structure})}{p(\text{seq})} \\
&\quad = \underset{(\text{seq}_{t_1},\ldots,\text{seq}_{t_n})\in S}{\operatorname{argmax}} \ \frac{\prod_{i=1}^{n} p(\text{seq}_{t_i}|\text{seq}_{t_0:t_{i-1}}, \text{structure})}{\prod_{i=1}^{n} p(\text{seq}_{t_i}|\text{seq}_{t_0:t_{i-1}})} \\
&\quad = \underset{(\text{seq}_{t_1},\ldots,\text{seq}_{t_n})\in S}{\operatorname{argmax}} \ \prod_{i=1}^{n} \frac{p(\text{seq}_{t_i}|\text{seq}_{t_0:t_{i-1}}, \text{structure})}{p(\text{seq}_{t_i}|\text{seq}_{t_0:t_{i-1}})}
\end{aligned}$$

where $\{t_i\}_{i=1}^{n}$ is a decoding order chosen by the user. Any position in the sequence can be held fixed, where seq_i is assigned, the i-th term in the product passes out of the argmax operator, and the subsequent tokens in the decoding order are conditioned on seq_i.

In NanoLuc experiments, residues essential to the protein function are fixed. In this case we decode using a ”proximity” decode order in order to prioritize selection of residues that stabilize the active site. We decode using beam search ($n = 128$ beams).

In WW experiments, we decode with an ”N-to-C” decode order, where residues are decoded in order from N-terminus to C-terminus. In this case we decode with greedy search.

A.2.3 Numerical Trick

Because small probabilities in the denominator can result in large probability ratios, we add a small quantity τ $(= 0.002)$ to the numerator and denominator probabilities, calculate the probability ratio, and re-normalize to sum to one. We find that this helps to avoid selecting residues with very low $p(\text{seq})$ (see Figure S10).

A.3 Current sequence design objectives

For comparison, we examine two sequence design objectives commonly used in the current literature.

A.3.1 $\underset{s \in S}{\operatorname{argmin}} \quad G(\mathbf{structure} = X, \mathbf{seq} = s)$

This is the objective used when designing a sequence that minimizes the Rosetta energy function [Alford et al., 2017]. Sequences designed via this objective often have rugged folding landscapes, and while the sequence is the minimum-energy sequence for the structure, the structure may not be the minimum-energy structure for the sequence [Norn et al., 2021].

A.3.2 $\underset{s \in S}{\operatorname{argmax}} \quad p(\mathbf{seq} = s | \mathbf{structure} = X)$ OR $\underset{s \in S}{\operatorname{argmax}} \quad p(\mathbf{seq} = s, \mathbf{structure} = X)$

First, we note that maximizing $p(\text{seq}, \text{structure} = X)$ over sequences is equivalent to maximizing $p(\text{seq}|\text{structure} = X)$:

$$
\begin{aligned}
&\underset{s \in S}{\operatorname{argmax}} \quad p(\text{seq} = s, \text{structure} = X) \\
&\quad = \underset{s \in S}{\operatorname{argmax}} \quad p(\text{seq} = s|\text{structure} = X) * \\
&\qquad p(\text{structure} = X) \\
&\quad = \underset{s \in S}{\operatorname{argmax}} \quad p(\text{seq} = s|\text{structure} = X)
\end{aligned}
$$

[Norn et al., 2021] discusses the importance of optimizing $p(\text{structure} = x|\text{seq})$, but the objective they use in practice is an objective that maximizes $p(\text{seq}, \text{structure} = X)$, factorized into $p(\text{structure} = X|\text{seq})$ and $p(\text{seq})$. The added $p(\text{seq})$ term is intended to minimize the divergence of designed sequences from known sequences. However, it voids the theoretical implications of maximizing $p(\text{structure} = X|\text{seq})$ (namely, maximizing Boltzmann probability and by extension protein stability and/or conformational specificity).

[Anishchenko et al., 2021] maximizes a $p(\text{seq}, \text{structure})$ objective similar to [Norn et al., 2021], but does not condition on a structure X.

[Dauparas et al., 2022] maximizes $p(\text{seq}|\text{structure} = X)$ directly, training an autoregressive model to predict protein sequence, conditioned on structure.

Similar to [Dauparas et al., 2022], several other state-of-the-art protein design algorithms involve a sequence decoder trained to maximize $p(\text{seq}|\text{structure})$ [Watson et al., 2022] [Ingraham et al., 2022].

B Experimental methods

B.1 NanoLuc synthesis and assays

B.1.1 DNA design

The NanoLuc protein sequence referred to as wild type in this work is the NanoLuc (PDB: 5IBO) amino acid sequence with an added N-terminal Strep

tag as shown in Supplemental Figure S6. DNA sequences to express wild type NanoLuc and the BayesDesign mutants were designed to include the following regions: Forward primer binding site, non-transcribed spacer, T7 promoter, Ribosome Binding Site, protein-coding region, T7 terminator, non-transcribed spacer, reverse primer binding site. The DNA protein-coding region for each mutant and the wild type control were generated from the amino acid sequences of each mutant and the wild type control. The DNA codon choice was optimized for E coli (ThermoFisher GeneArt) and DNA gene fragments were constructed by Twist Bioscience (San Francisco, CA) and amplified by Q5 PCR (NEB, Ipswich, MA, USA).

B.1.2 Cell-free protein synthesis

Cell-free protein synthesis (CFPS) was conducted as described in [Hunt et al., 2022]. Cell extract was prepared using BL21-Star™ (DE3) E coli cells (Invitrogen, Carlsbad, CA) cultured in 2xYT media. The cells were induced with isopropyl β-d-1-thiogalactopyranoside (IPTG) at OD600 0.5 to 0.7, harvested at OD600 of 2 to 4, washed, and lysed with 3 passes through an Avestin Emulsiflex B-15 homogenizer (Avestin, Ottawa, Canada) at 21000 psi. Lysate was centrifuged at 12000 RCF for 30 minutes and the supernatant was harvested as cell extract, which is used at 25% (v/v) in CFPS reactions. For DNA template in CFPS reactions, unpurified PCR product was added at 33% (v/v). A PANOx-SP mixture of nucleotides, amino acids, energy substrates and other small molecules was added using reaction concentrations described in [Jewett and Swartz, 2004]. For yield determination, 2 μL samples of unpurified CFPS reaction product were dried on filter paper, precipitated and washed with TCA, and measured with a scintillation counter. Yields were calculated based on the percentage of incorporated 14C-leucine, which was present at a concentration of 5μM in the initial reaction preparation [Bundy and Swartz, 2010].

B.1.3 Identification of active site

Using Amber's ff14SB force field and OpenMM's molecular dynamics engine, we ran energy minimization on the NanoLuciferase crystal structure (5IBO). Docking runs were then performed with Vina, LeDock, and Plants 1.2, using the minimized protein and its ligand, furimazine. The area explored by the docking software consisted of a box that encompassed the entire beta barrel structure of the enzyme, based on the description of the active site in [Tomabechi et al., 2016]. Three MD simulations were then performed to verify that furimazine remained in the docking active site, using the best docked pose from Vina as the starting structure. OpenFF's SMIRNOFF force field was used to parameterize furimazine, and Amber's ff14SB force field was used to parameterize the protein. Solvation and charge neutralization, minimization, NPT equilibration, and the 100 ns simulations were all carried out with OpenMM. Chimera was then used to identify amino acid residues within 5Åof the consensus docking poses. For the large active site, all residues within 7Åwere selected. These amino acids

were presumed to comprise the active site of the protein.

B.1.4 NanoLuc Heat Treatment, Solubility Assessment, and Activity Assay

The NanoLuc proteins were expressed as 3 biological replicates of 50 μL CFPS reactions for each mutant in parallel. Reactions were incubated for 3 hours at 37° C and 280 RPM. Each reaction replicate was sampled for scintillation counting yield and redivided into five aliquots in PCR tubes, where each aliquot was heat treated within the range of $37°C$ and $95°C$. Heat treatment was conducted for 15 minutes in a PCR thermocycler chamber, or an incubator for the 37° C heat treatment. After heat treatment, all samples were centrifuged for 15 min at 16100 g at 4° C and the supernatant was sampled in triplicate for scintillation counting to assess soluble NanoLuc protein remaining after heat treatment.

B.2 WW synthesis and assays

B.2.1 Synthesis

WW, BayesDesign and ProteinMPNN peptides were synthesized as C-terminal acids, by microwave-assisted solid-phase peptide synthesis, using a standard Fmoc Nα protection strategy. Amino acids were activated by 2-(1H-benzotriazole-1-yl)-1,1,3,3-tetramethyluronium hexafluorophosphate (HBTU, purchased from Advanced ChemTech) and N-hydroxybenzotriazole hydrate (HOBt, purchased from Advanced ChemTech). Fmoc-Gly-loaded Novasyn Wang resin and all Fmoc-protected α-amino acids with acid-labile side-chain protecting groups were purchased from CombiBlocks. Peptide variants were synthesized on a 50 μmol scale. Acid-labile side-chain protecting groups were globally removed and proteins were cleaved from the resin by stirring the resin for 4h in a solution of phenol (0.125 g), water (125 μL), thioanisole (125 μL), ethanedithiol 62.5 μL) and triisopropylsilane (25 μL) in trifluoroacetic acid (TFA, 2 mL). Following the cleavage reaction, the TFA solution was drained from the resin, the resin was rinsed with additional TFA. Proteins were precipitated from the concentrated TFA solution by addition of diethyl ether (40 mL). Following centrifugation, the ether was decanted, and the pellet was dissolved in 40mL 1:1 H2O/MeCN, frozen and lyophilized to remove volatile impurities. The resulting powder was stored at -20° C until purification.

B.2.2 Purification and Characterization

Immediately prior to purification, the crude protein was dissolved in 1:1 H2O/MeCN. Proteins were purified by preparative reverse-phase HPLC on a C18 column using a linear gradient of water in acetonitrile with 0.1% v/v TFA. HPLC fractions containing the desired protein product were pooled, frozen, and lyophilized. Proteins were identified by electrospray ionization time of flight mass spectrom-

etry (ESI-TOF, values shown in table 4 below), and purity was analyzed by Analytical HPLC (see Figure 16).

B.2.3 Circular Dichroism Spectropolarimetry

Measurements were made with an Aviv 420 Circular Dichroism (CD) Spectropolarimeter, using quartz cuvettes with a path length of 0.1 cm. Protein solutions were prepared in 20 mM sodium phosphate buffer, pH 7, and protein concentrations were determined spectroscopically based on tyrosine and tryptophan absorbance at 280 nm in 6 M guanidine hydrochloride + 20 mM sodium phosphate (ϵTrp = 5690 M-1 cm-1 , ϵTyr = 1280 M-1 cm1). CD spectra of 50 μM solutions were obtained from 260 to 200 nm at 25° C and 95° C.

B.2.4 Molecular Dynamics and CD prediction

For further validation of structures corresponding to observed CD spectra, we ran energy minimization on the AlphaFold-predicted structures of the wild type, BayesDesign, and ProteinMPNN sequences. We used Amber's ff14SB force field and OpenMM's molecular dynamics (MD) engine. Solvation and charge neutralization, minimization, NPT equilibration, and the 100 ns simulations were all carried out with OpenMM. We then used the PDBMD2CD tool [Drew and Janes, 2020] to predict CD spectra for 600 evenly-space frames of each MD simulation. We selected the predicted CD with lowest Mean Absolute Error compared to the observed CD spectra, and considered the corresponding MD frames to be the structures corresponding to the observed CD.

C Proofs

Proposition 1. *If a sequence s maximizes stability, then it maximizes the probability ratio between the folded and unfolded states.*

Proof: Consider two sequences s and s', and assume

$$G(Z'|\text{seq} = s') - G(X'|\text{seq} = s') < G(Z|\text{seq} = s) - G(X|\text{seq} = s).$$

Note that e^{-x} is a monotonically decreasing function, and for monotonically decreasing functions, $a < b \implies f(a) > f(b)$. Thus:

$$e^{-(G(Z'|\text{seq}=s')-G(X'|\text{seq}=s'))} > e^{-(G(Z|\text{seq}=s)-G(X|\text{seq}=s))}$$

$$\frac{p(\text{structure} = Z'|\text{seq} = s')}{p(\text{structure} = X'|\text{seq} = s')} > \frac{p(\text{structure} = Z|\text{seq} = s)}{p(\text{structure} = X|\text{seq} = s)}$$

Since both sides of the inequality are positive,

$$\frac{p(\text{structure} = X'|\text{seq} = s')}{p(\text{structure} = Z'|\text{seq} = s')} < \frac{p(\text{structure} = X|\text{seq} = s)}{p(\text{structure} = Z|\text{seq} = s)}$$

□

D Supplemental figures

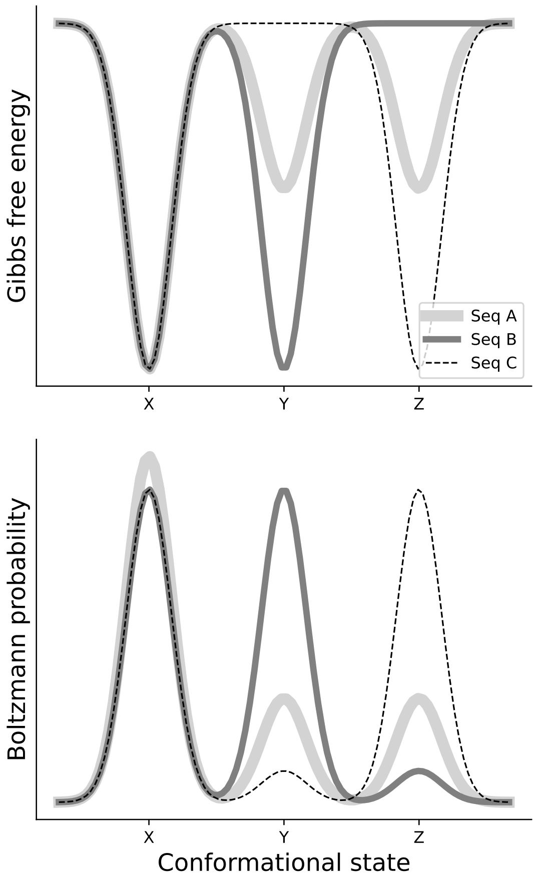

Figure 1: Protein sequences can have equal Gibbs free energy for a given structure, while having varying Boltzmann probability, stability, and conformational specificity, depending on the Gibbs free energy of other states. X, Y, and Z denote the native state, the alternate folded state, and the denatured state, respectively.

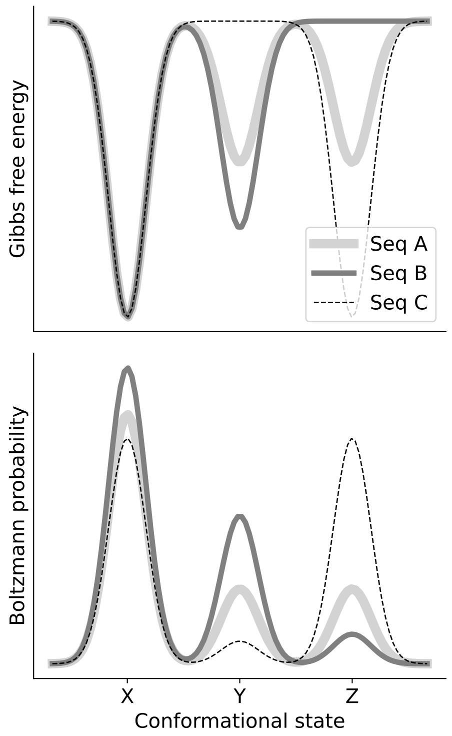

Figure 2: A case (i.e. a set of energy landscapes) where optimizing Boltzmann probability would result in the maximum stability sequence.

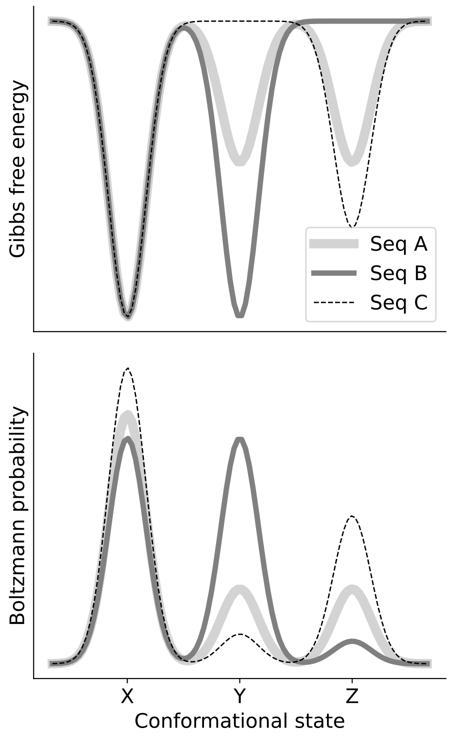

Figure 3: A case (i.e. a set of energy landscapes) where optimizing Boltzmann probability would result in the maximum specificity sequence.

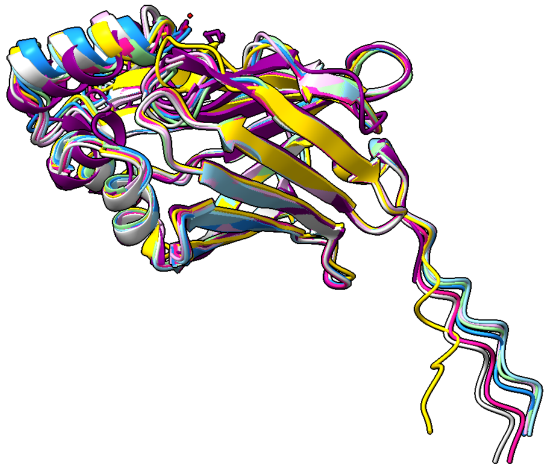

Figure 4: Structure alignment of the Alphafold-predicted structures of all mutants with the WT predicted structure.

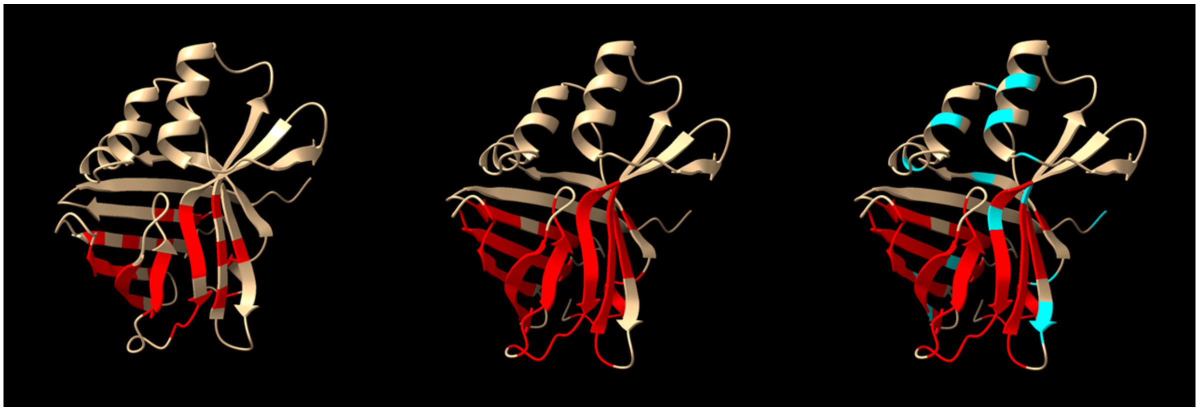

Figure 5: Several regions of the NanoLuc enzyme were considered in enzyme redesign. On the left, red highlights the residues around the enzyme active site. In the middle, the (red) region around the active site is expanded to include more residues. On the right, 14 previously hand-engineered residues are also highlighted in cyan. For our four mutants, we applied four design variations: 1) holding only the small active site amino acids fixed and designing all others (B1/M1), 2) holding the the small active site amino acids and the engineered residues fixed and designing all others (B2/M2), 3) holding only the large active site amino acids fixed and designing all others (B3/M3), and 4) holding the the large active site amino acids and the engineered residues fixed and designing all others (B4/M4).

```
PDB_5IBO      -----SDNMVFTLEDFVGDWRQTAGYNLDQVLEQGGVSSLFQNLGVSVTPIQRIVLSGEN     55
Wild_Type     MWSHPQFEKVFTLEDFVGDWRQTAGYNLDQVLEQGGVSSLFQNLGVSVTPIQRIVLSGEN     60
                   . : ***************************************************

PDB_5IBO      GLKIDIHVIIPYEGLSGDQMGQIEKIFKVVYPVDDHHFKVILHYGTLVIDGVTPNMIDYF     115
Wild_Type     GLKIDIHVIIPYEGLSGDQMGQIEKIFKVVYPVDDHHFKVILHYGTLVIDGVTPNMIDYF     120
              ************************************************************

PDB_5IBO      GRPYEGIAVFDGKKITVTGTLWNGNKIIDERLINPDGSLLFRVTINGVTGWRLCERILA      174
Wild_Type     GRPYEGIAVFDGKKITVTGTLWNGNKIIDERLINPDGSLLFRVTINGVTGWRLCERILA      179
              ***********************************************************
```

Figure 6: Clustal Omega multiple sequence alignment of the NanoLuc sequence referred to as wild type (WT) in this paper to the sequence of PDB 5IBO.

		Bayes				MPNN			
		B1	B2	B3	B4	M1	M2	M3	M4
Bayes	B1								
	B2	64							
	B3	65	67						
	B4	59	74	81					
MPNN	M1	62	56	60	60				
	M2	59	74	66	72	73			
	M3	59	65	77	75	73	73		
	M4	58	69	77	86	66	83	83	
	WT	48	60	67	72	49	60	66	71

Figure 7: A homology matrix comparing the extent of amino acid percent sequence identity of the NanoLuc sequences studied in this work.

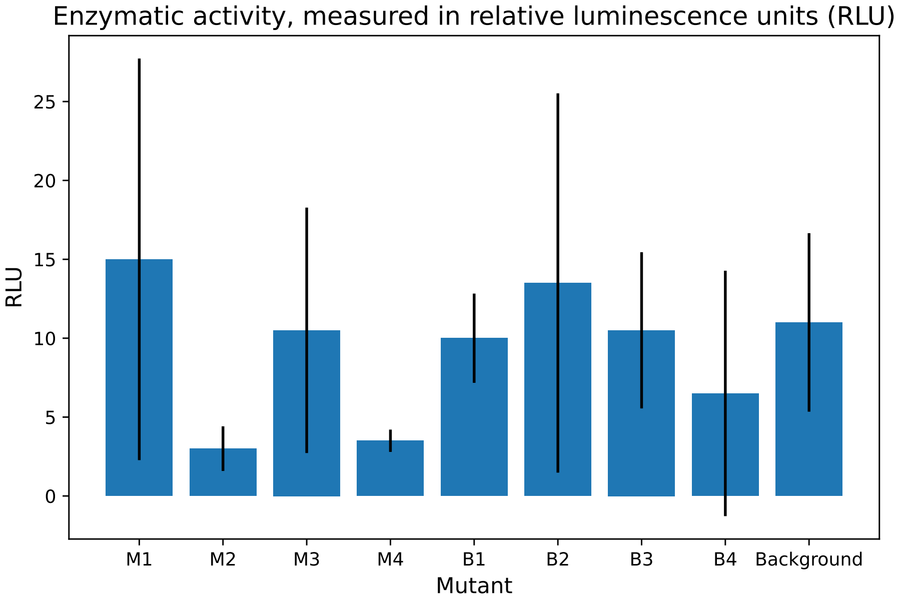

Figure 8: NanoLuc activity assay results for BayesDesign (B1-B4) and Protein MPNN (M1-M4) sequences measured in relative luminescent units (RLU). The enzymatic activity for these mutants is negligible.

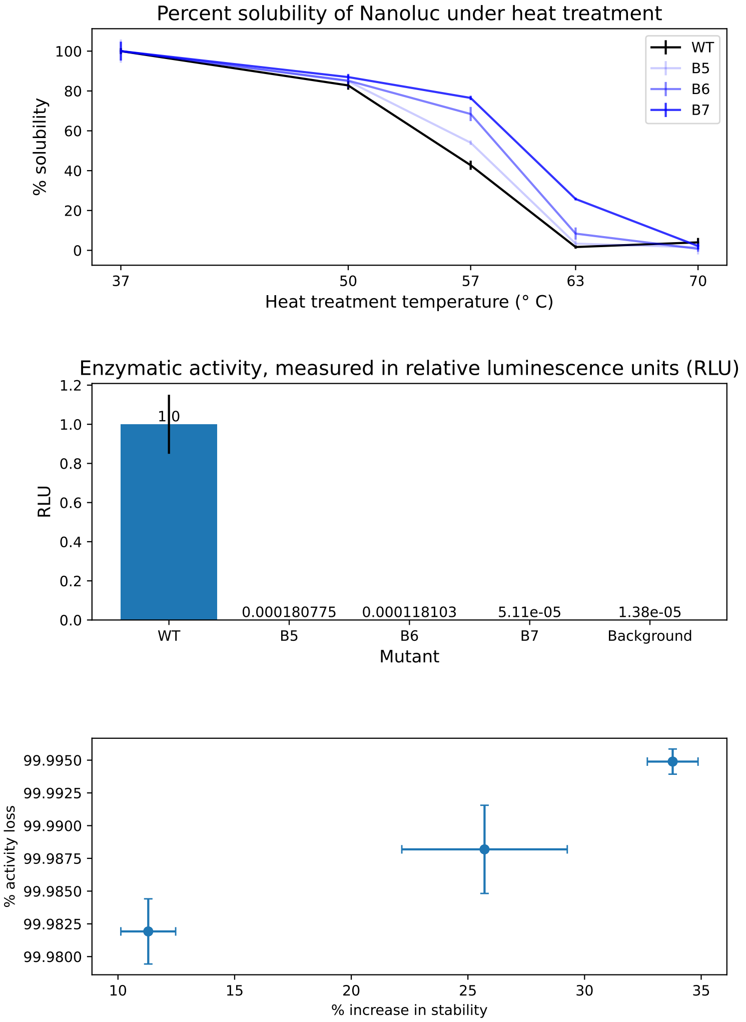

Figure 9: Thermal stability and enzymatic activity of wild type NanoLuc compared to BayesDesign NanoLuc mutants B5, B6, and B7, which have two amino acid substitution mutations. Each has the Q52Y mutation and B5, B6, and B7 have the unique mutations R53H, Y91E, and V48T, respectively. A) Each protein was expressed in a cell-free protein synthesis system and parallel aliquots were subjected to 15-minute heat treatments at 37, 50, 57, 63, or 70°C and the remaining soluble protein was measured. The solubility data is the average of n = 3 biological replicates and the standard deviation error bars are shown as vertical lines. Interpolating lines show the temperature-dependent stability trend for each protein mutant. B) The enzymatic activity of the BayesDesign mutants B5, B6, and B7 compared to the wild type enzyme. C) Enzymatic activity loss correlated with increased stability for BayesDesign mutants B5, B6 and B7. The enzymatic activity is reported relative to wild type NanoLuc and the stability data is also relative to wild type NanoLuc after 57° C heat treatment.

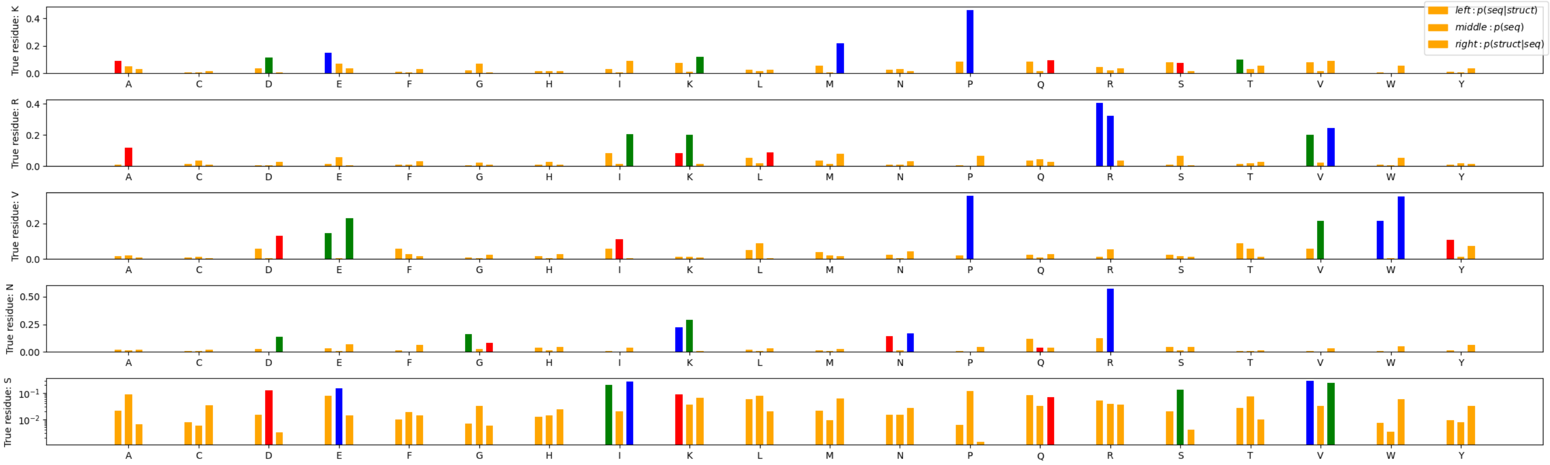

Figure 10: A visualization of probability shift induced by applying Bayes' rule to ProteinMPNN probabilities. Each row represents an evenly spaced residue in the WW peptide. Each column represents an amino acid. The first bar in each cluster corresponds to $p(\text{seq}|\text{structure})$; the second, to $p(\text{seq})$, and the third to $p(\text{seq}|\text{structure})/p(\text{seq})$, normalized across amino acids. The top score for each model is in blue, the second highest is in red, and the third highest is in green. In the second row, ProteinMPNN's best guess is arginine, but XLNet is in agreement, so the third model ends up choosing valine, which was originally the third choice of ProteinMPNN.

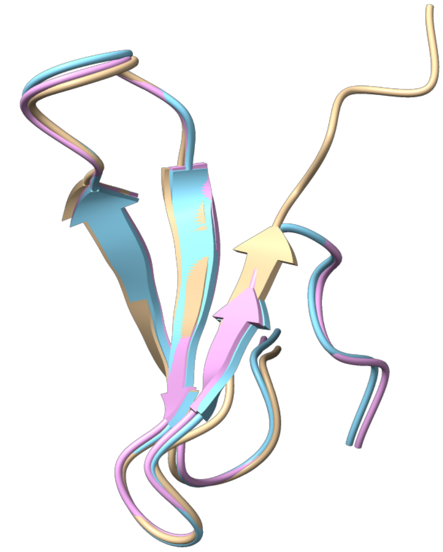

Figure 11: AlphaFold-predicted structures for the WW wild type (magenta), BayesDesign (tan), and ProteinMPNN (cyan) sequences.

Table 1: Wild type and designed sequences for NanoLuc. Masked positions are designed.

WT<br>Sequence	MWSHPQFEKVFTLEDFVGDWRQTAGYNLDQVLEQGGVSSLFQNLGVSVTPIQRIVLSG<br>ENGLKIDIHVIIPYEGLSGDQMGQIEKIFKVVYPVDDHHFKVILHYGTLVIDGVTPNM<br>IDYFGRPYEGIAVFDGKKITVTGTLWNGNKIIDERLINPDGSLLFRVTINGVTGWRLC<br>ERILA
B1/M1<br>Masked sequence	MWSHPQFEKV--L-------------------------------------I-------<br>E-G-K------------------------------------VIL-Y------------<br>--<br>-----
BayesDesign - B1	MWSHPQFEKVLTLDDFVGNWRMVSQWNIPAVLREMGMPPFLIDLWCATTPIWVITKYG<br>ENGLKVDVHMVIPKEGLTPEQMRYLQAMFGHMTQVDETHFQVILDYGVFIINGTSKNC<br>KDFMNRPFEVNTTFDGKKLTMTGTLWNGKKFVMTFEILPDGHLRYTVDVNGVKGWMIL<br>ERVEP
ProteinMPNN - M1	MWSHPQFEKVLTLDDFVGKWEEVEKKNMAEVLKEMGLPEFLIELYLKTKPILVIEKSG<br>ENGLKVTVELIIPKEGLTEEQIKEIEKIFKKIEKVDENNFKVILDYGTLIVNGKSENM<br>KDFLNKPFTGNATFDGKTLTVTGTLSNGKKVTITFTILPDGSLKLTIEVNGVKGEMIL<br>KKIEE
B2/M2<br>Masked sequence	MWSHPQFEKV--LE------R------L--------V-----N-------I-RI----<br>E-G-KI-------------D---Q--K--------------VIL-Y-T-----V-P--<br>----GRPYE-I----G-KITV------G-K-I-------D-S-L--------------<br>-R---
BayesDesign - B2	MWSHPQFEKVLKLEDFVGDWRRVDSWNLPEVLKAMGVPQFFINLFCQTQPIWRISKHG<br>EKGLKIQMIMRIPKQGLTPDQMAQIQKTFKHVQDIDDQHFQVILDYGTLIIDGVSPNC<br>KDFLGRPYEGICKFDGKKITVTGTLPNGNKFIWTMEILDDGSLLFTVDVNGVKGYMIL<br>ERVEP
ProteinMPNN - M2	MWSHPQFEKVLTLEDFVGDWREVKKENLAEVLKEMGVPEFFINLFLNTKPILRIEKAG<br>ENGLKITIELIIPKKGLTKDQLEQIKKIFKKVEEIDENNFKVILDYGTLIINGVSPNM<br>KDFLGRPYEGIATFDGKKITVTGTLPDGRKVIITFEILEDGSLLLTVEVNGVKGSMIL<br>ERVEK
B3/M3<br>Masked sequence	MWSHPQFEKVFTLE--V-------------------------------TPI-----GE<br>NGLKI---------------------------------FKVILHYGTLV-DGVTPNM-<br>--FGRPYEGI----GKKITVTG--WNGNKIIDE-----DGS-LFR-------------<br>----
BayesDesign - B3	MWSHPQFEKVFTLEDFVGDWRLVSKQNMAAVLREMGAPDFLIQLYLQCTPIFHITKSG<br>ENGLKIDVEMIIPKAGLTPEQMCYLQKMFKHMEPVDENHFKVILHYGTLVIDGVTPNM<br>KDAFGRPYEGICKFDGKKITVTGTLWNGNKIIDEYEILPDGSLLFRRTVNGVTGWMKL<br>ERVEP
ProteinMPNN - M3	MWSHPQFEKVFTLEDFVGKWKKVSSKNIKEVLKEMGAPEFLIELFEKTTPILNITKSG<br>ENGLKIDIELIIPKEGLTEEQIKEIEKIFKKIEKVDENNFKVILHYGTLVIDGVTPNM<br>KDFFGRPYEGIAKFDGKKITVTGTLWNGNKIIDEYEILEDGSLLFRVTVNGVKGEMIL<br>EKIEE

Table 2: Wild type and designed sequences for NanoLuc (continued). Masked positions are designed. B5-B7 were designed by an algorithm whereby, out of all of the masked positions, the position for which the BayesDesign probability exceeded the wild type probability by the greatest amount was selected for redesign.

B4/M4	
Masked sequence	MWSHPQFEKVFTLE--V---R------L--------V-----N-----TPI-RI--SG<br>ENGLKI-------------D---Q--K------------FKVILHYGTLV-DGVTPNM<br>---FGRPYEGI----GKKITVTG--WNGNKIIDE-----DGS-LFR------------<br>-R---
BayesDesign - B4	MWSHPQFEKVFTLEDFVGDWREVDRWNLADVLKAMGVPQFLINLYMSCTPIWRITKSG<br>ENGLKIDVEMIIPKQGLTEDQLQQIKKIFQHVEDVDDNHFKVILHYGTLVIDGVTPNM<br>KDWFGRPYEGICKFDGKKITVTGTLWNGNKIIDEFEILPDGSLLFRTTVNGVTGYRIL<br>ERVEP
ProteinMPNN - M4	MWSHPQFEKVFTLEDFVGDWREVKRENLAEVLKAMGVPEFLINLYLKTTPILRIEKSG<br>ENGLKIDIELIIPKEGLTEDQLEQIKKIFGKLEEVDENNFKVILHYGTLVIDGVTPNM<br>KDFFGRPYEGIAKFDGKKITVTGTLWNGNKIIDEFEILEDGSLLFRVTVNGVTGSRIL<br>ERVEK
B5	
BayesDesign - B5	MWSHPQFEKVFTLEDFVGDWRQTAGYNLDQVLEQGGVSSLFQNLGVSVTPIYHIVLSG<br>ENGLKIDIHVIIPYEGLSGDQMGQIEKIFKVVYPVDDHHFKVILHYGTLVIDGVTPNM<br>IDYFGRPYEGIAVFDGKKITVTGTLWNGNKIIDERLINPDGSLLFRVTINGVTGWRLC<br>ERILA
BayesDesign - B6	MWSHPQFEKVFTLEDFVGDWRQTAGYNLDQVLEQGGVSSLFQNLGVSVTPIYRIVLSG<br>ENGLKIDIHVIIPYEGLSGDQMGQIEKIFKVVEPVDDHHFKVILHYGTLVIDGVTPNM<br>IDYFGRPYEGIAVFDGKKITVTGTLWNGNKIIDERLINPDGSLLFRVTINGVTGWRLC<br>ERILA
B7	
BayesDesign - B7	MWSHPQFEKVFTLEDFVGDWRQTAGYNLDQVLEQGGVSSLFQNLGVSTTPIYRIVLSG<br>ENGLKIDIHVIIPYEGLSGDQMGQIEKIFKVVYPVDDHHFKVILHYGTLVIDGVTPNM<br>IDYFGRPYEGIAVFDGKKITVTGTLWNGNKIIDERLINPDGSLLFRVTINGVTGWRLC<br>ERILA

Table 3: Wild type and designed sequences for WW. Masked positions are designed.

WT	
Sequence	KLPPGWEKRMSRSSGRVYYFNHITNASQFERPSG
ProteinMPNN	
Masked sequence	---------------------------------G
Designed sequence	TLPEGWVEVVDPETGEKKYYNTKTKEVTSEKPVG
BayesDesign	
Masked sequence	---------------------------------G
Designed sequence	TLPEHWVKRKDPKTGQWIYENTKTHETLAQKWQG

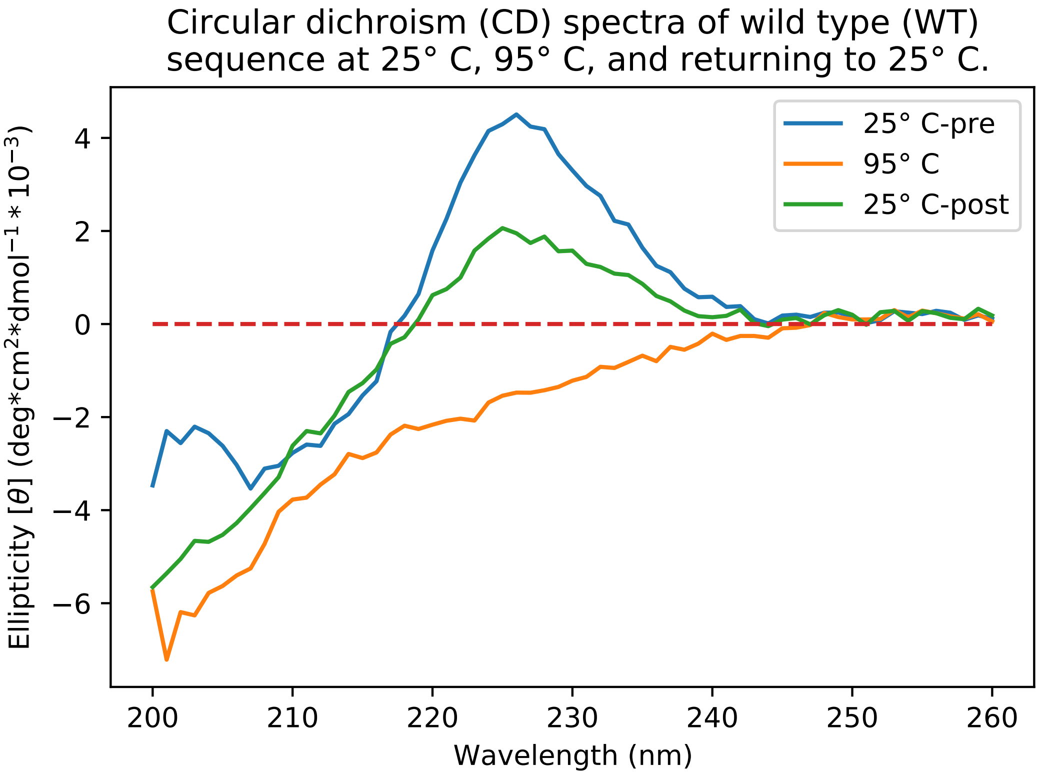

Figure 12: WW has a characteristic peak at ∼227nm, and has low reversibility of 56% - it fails to recover its original CD spectrum after heat treatment and cooling.

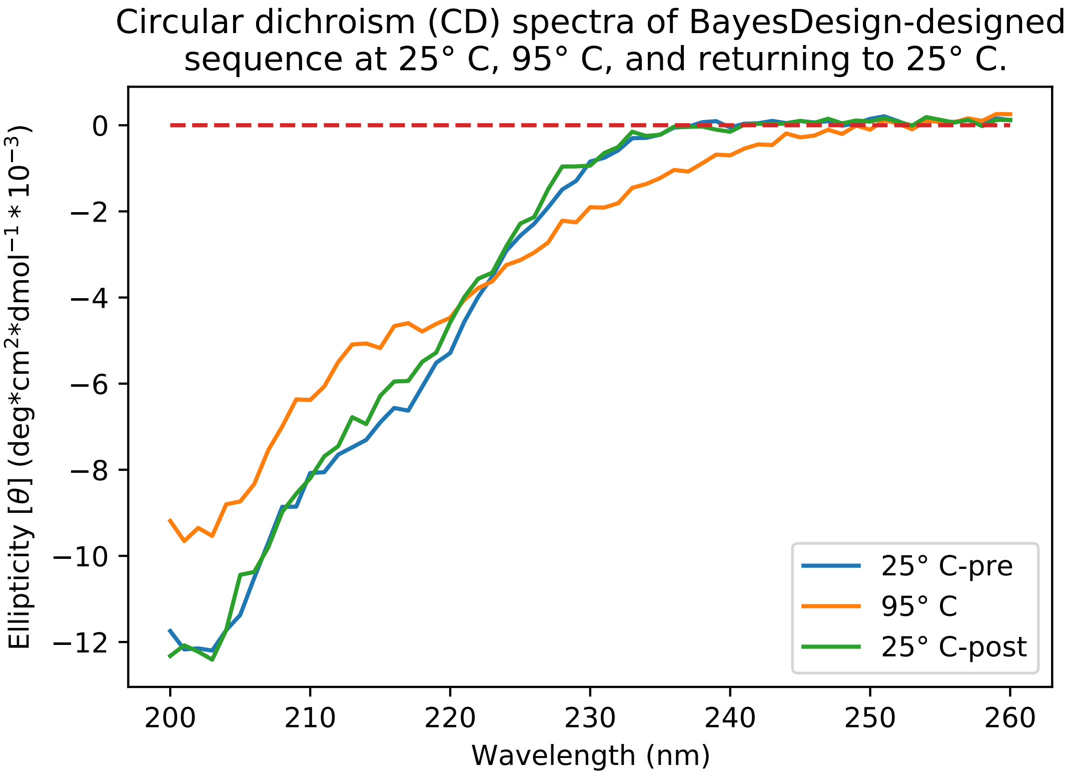

Figure 13: Although the BayesDesign design for WW does not follow the WT CD profile exactly, its profile shifts when heated and regains its original profile when returned to 25° C. This suggests that the designed sequence adopts its folded conformation with high conformational specificity.

Table 4: Mass spectrum data for synthesized WW mutants

Peptide Name	m/z	Expected Mass	Observed Mass
Wild type	4	996.2513	996.2494
ProteinMPNN	3	1279.9868	1279.9804
BayesDesign	3	1378.7160	1378.7075

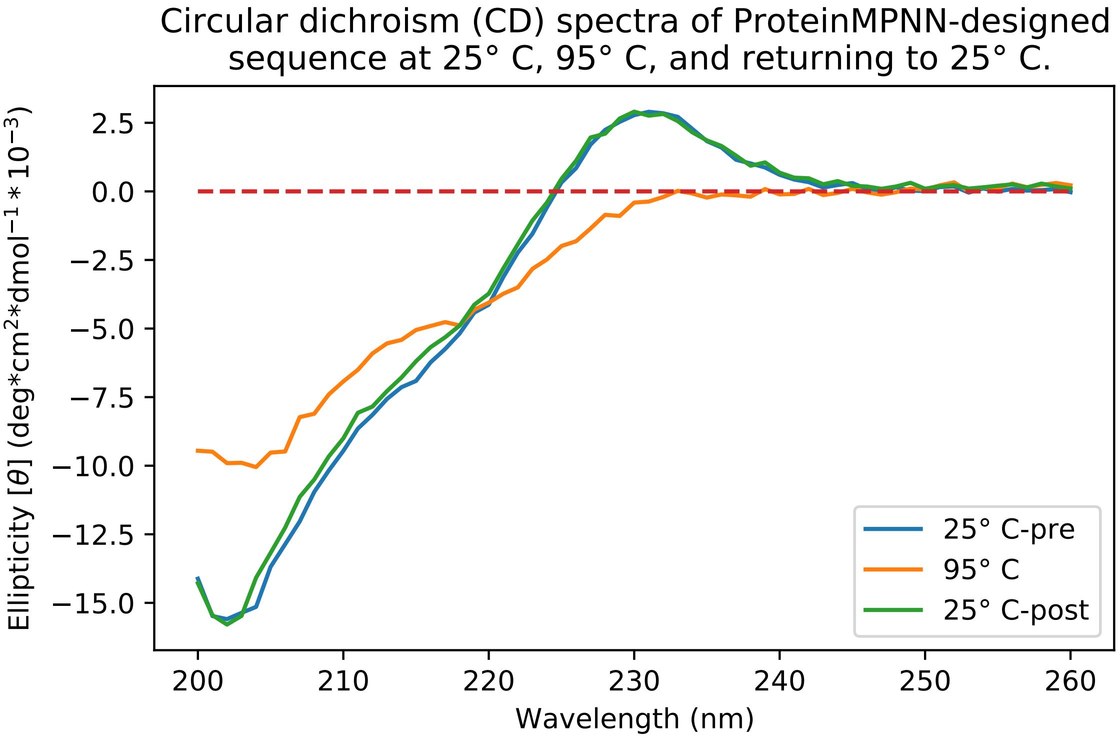

Figure 14: Although the ProteinMPNN design for WW does not follow the WT CD profile exactly, its profile shifts when heated and regains its original profile when returned to 25° C. This suggests that the designed sequence adopts its folded conformation with high conformational specificity.

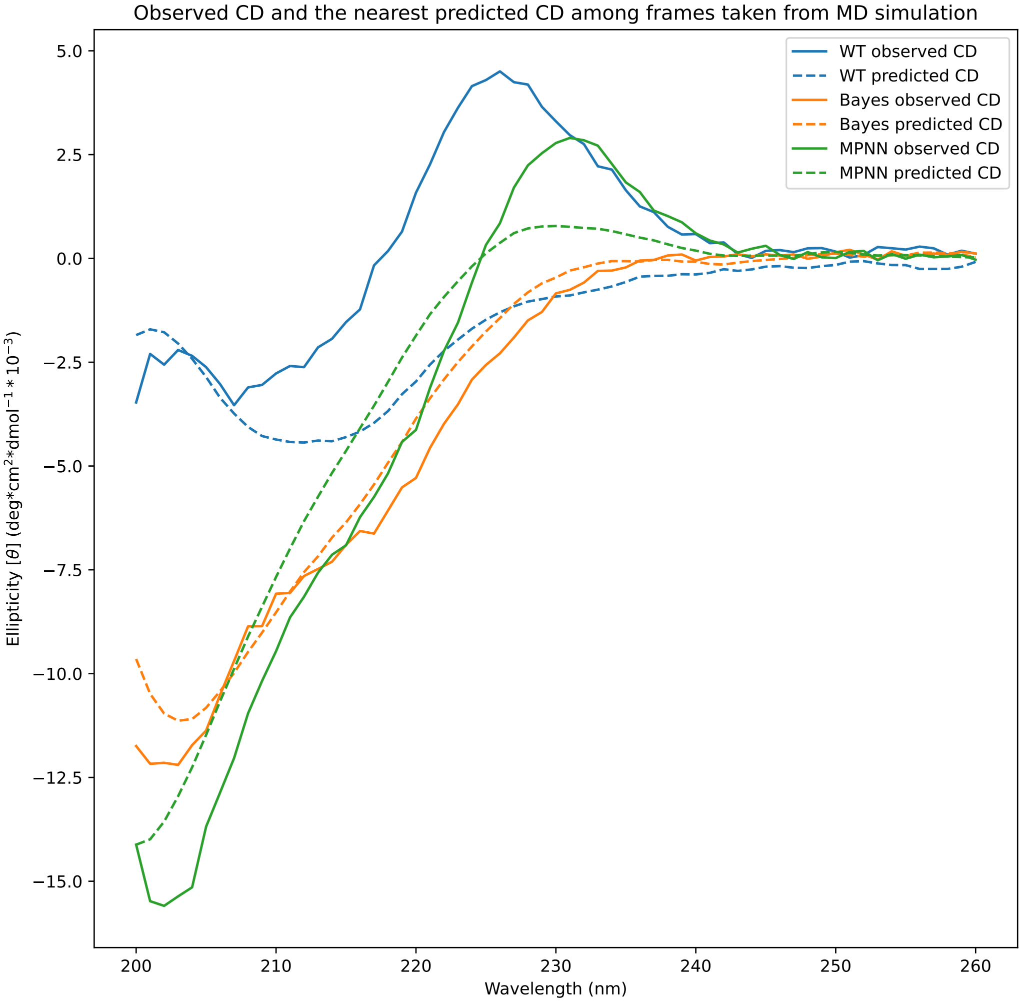

Figure 15: To evaluate how closely the BayesDesign and ProteinMPNN structures matched the wild type WW conformation, we used the PDBMD2CD tool to predict spectra for frames from a molecular dynamics simulation. The closest-matching spectra, plotted here, were used to obtain structures for Figure 5 in the main text.

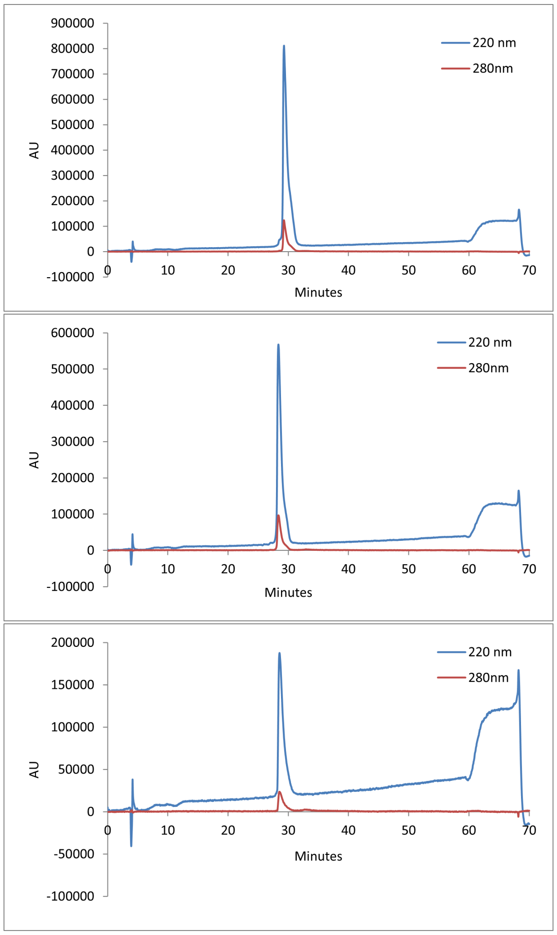

Figure 16: Analytical HPLC trace for WW wild type (top), BayesDesign (middle), and ProteinMPNN (bottom).

References

[Alford et al., 2017] Alford, R. F., Leaver-Fay, A., Jeliazkov, J. R., O'Meara, M. J., DiMaio, F. P., Park, H., Shapovalov, M. V., Renfrew, P. D., Mulligan, V. K., Kappel, K., Labonte, J. W., Pacella, M. S., Bonneau, R., Bradley, P., Dunbrack, R. L., Das, R., Baker, D., Kuhlman, B., Kortemme, T., and Gray, J. J. (2017). The rosetta all-atom energy function for macromolecular modeling and design. *Journal of Chemical Theory and Computation*, 13(6):3031–3048.

[Anishchenko et al., 2021] Anishchenko, I., Pellock, S. J., Chidyausiku, T. M., Ramelot, T. A., Ovchinnikov, S., Hao, J., Bafna, K., Norn, C., Kang, A., Bera, A. K., DiMaio, F., Carter, L., Chow, C. M., Montelione, G. T., and Baker, D. (2021). De novo protein design by deep network hallucination. *Nature*, 600(7889):547–552.

[Bundy and Swartz, 2010] Bundy, B. C. and Swartz, J. R. (2010). Site-specific incorporation of p-propargyloxyphenylalanine in a cell-free environment for direct protein-protein click conjugation. *Bioconjugate Chemistry*, 21(2):255–263. PMID: 20099875.

[Dauparas et al., 2022] Dauparas, J., Anishchenko, I., Bennett, N., Bai, H., Ragotte, R. J., Milles, L. F., Wicky, B. I. M., Courbet, A., de Haas, R. J., Bethel, N., Leung, P. J. Y., Huddy, T. F., Pellock, S., Tischer, D., Chan, F., Koepnick, B., Nguyen, H., Kang, A., Sankaran, B., Bera, A. K., King, N. P., and Baker, D. (2022). Robust deep learning based protein sequence design using proteinmpnn. *bioRxiv*.

[Drew and Janes, 2020] Drew, E. D. and Janes, R. W. (2020). PDBMD2CD: providing predicted protein circular dichroism spectra from multiple molecular dynamics-generated protein structures. *Nucleic Acids Research*, 48(W1):W17–W24.

[Elnaggar et al., 2020] Elnaggar, A., Heinzinger, M., Dallago, C., Rihawi, G., Wang, Y., Jones, L., Gibbs, T., Feher, T., Angerer, C., Steinegger, M., Bhowmik, D., and Rost, B. (2020). Prottrans: Towards cracking the language of life's code through self-supervised deep learning and high performance computing.

[Hunt et al., 2022] Hunt, J. P., Zhao, E. L., Free, T. J., Soltani, M., Warr, C. A., Benedict, A. B., Takahashi, M. K., Griffitts, J. S., Pitt, W. G., and Bundy, B. C. (2022). Towards detection of sars-cov-2 rna in human saliva: A paper-based cell-free toehold switch biosensor with a visual bioluminescent output. *New Biotechnology*, 66:53–60.

[Ingraham et al., 2022] Ingraham, J., Baranov, M., Costello, Z., Frappier, V., Ismail, A., Tie, S., Wang, W., Xue, V., Obermeyer, F., Beam, A., and Grigoryan, G. (2022). Illuminating protein space with a programmable generative model. *bioRxiv*.

[Jewett and Swartz, 2004] Jewett, M. C. and Swartz, J. R. (2004). Mimicking the escherichia coli cytoplasmic environment activates long-lived and efficient cell-free protein synthesis. *Biotechnology and Bioengineering*, 86(1):19–26.

[Norn et al., 2021] Norn, C., Wicky, B. I. M., Juergens, D., Liu, S., Kim, D., Tischer, D., Koepnick, B., Anishchenko, I., null null, Baker, D., and Ovchinnikov, S. (2021). Protein sequence design by conformational landscape optimization. *Proceedings of the National Academy of Sciences*, 118(11):e2017228118.

[Tomabechi et al., 2016] Tomabechi, Y., Hosoya, T., Ehara, H., ichi Sekine, S., Shirouzu, M., and Inouye, S. (2016). Crystal structure of nanokaz: The mutated 19 kda component of oplophorus luciferase catalyzing the bioluminescent reaction with coelenterazine. *Biochemical and Biophysical Research Communications*, 470(1):88–93.

[Watson et al., 2022] Watson, J. L., Juergens, D., Bennett, N. R., Trippe, B. L., Yim, J., Eisenach, H. E., Ahern, W., Borst, A. J., Ragotte, R. J., Milles, L. F., Wicky, B. I. M., Hanikel, N., Pellock, S. J., Courbet, A., Sheffler, W., Wang, J., Venkatesh, P., Sappington, I., Torres, S. V., Lauko, A., De Bortoli, V., Mathieu, E., Barzilay, R., Jaakkola, T. S., DiMaio, F., Baek, M., and Baker, D. (2022). Broadly applicable and accurate protein design by integrating structure prediction networks and diffusion generative models. *bioRxiv*.

[Zhu et al., 2015] Zhu, G.-F., Ren, S.-Y., Xi, L., Du, L.-F., and Zhu, X.-F. (2015). Temperature induced structural transitions from native to unfolded aggregated states of tobacco etch virus protease. *Journal of Molecular Structure*, 1082:80–90.
